# Genetic Diversity Analysis based on the Virulence, Physiology and Regional Variability in Different Isolates of Powdery Mildew in Pea

**DOI:** 10.3390/jof8080798

**Published:** 2022-07-29

**Authors:** Parthasarathy Seethapathy, Subbiah Sankaralingam, Deepu Pandita, Anu Pandita, Kousalya Loganathan, Shabir Hussain Wani, Diaa O. El-Ansary, Hanoor Sharma, Ryan Casini, Eman A. Mahmoud, Hosam O. Elansary

**Affiliations:** 1 Department of Plant Pathology, Amrita School of Agricultural Sciences, Amrita Vishwa Vidyapeetham, Coimbatore 642109, India; 2 PG and Research Department of Botany, Saraswathi Narayanan College, Madurai 625022, India; biosankaralingam@yahoo.co.in; 3 Government Department of School Education, Jammu 180001, India; 4 Vatsalya Clinic, Krishna Nagar, New Delhi 110051, India; dt.anunischal46@gmail.com; 5 Department of Botany, Nirmala College for Women, Coimbatore 641018, India; lkousalya25@gmail.com; 6 Mountain Research Centre for Field Crops, Sher-e-Kashmir University of Agricultural Sciences and Technology of Kashmir, Khudwani Anantnag 192101, Jammu and Kashmir, India; shabirhussainwani@gmail.com; 7 Precision Agriculture Laboratory, Department of Pomology, Faculty of Agriculture (El-Shatby), Alexandria University, Alexandria 21545, Egypt; diaaagri@hotmail.com; 8 Microbiology and Immunology Department, Wright State University, Dayton, OH 45435, USA; hanoor.sharma@clearlabs.com; 9 College of Public Health, University of California, Berkeley, 2121 Berkeley Way, Berkeley, CA 94704, USA; ryan.casini@berkeley.edu; 10 Department of Food Industries, Faculty of Agriculture, Damietta University, Damietta 34511, Egypt; emanmail2005@yahoo.com; 11 Plant Production Department, College of Food & Agriculture Sciences, King Saud University, Riyadh 11451, Saudi Arabia; helansary@ksu.edu.sa

**Keywords:** *Erysiphe pisi*, internal transcribed spacer, *Pisum sativum*, powdery mildew, random amplified polymorphic DNA

## Abstract

Powdery mildew is an omnipresent disease that reduces the yield and quality of pea crops (*Pisum sativum* L.). To examine the powdery mildew pathogen’s morphological, molecular, and genetic diversity, we collected samples of powdery mildew-affected pea crops from ten distinct locations in the Nilgiris district of Tamil Nadu, India. The pathogen *Erysiphe pisi* was identified morphologically based on anamorphic characters. Molecular identification of *E. pisi* isolates was befitted by targeting the internal transcribed spacer (ITS) region of rDNA and specific primers of powdery mildew fungi. The genetic variation between ten different *E. pisi* isolates collected from topographically distinct mountainous areas was studied using random amplified polymorphic (RAPD). Based on its morphological characteristics, the powdery mildew fungus presented high similarities to *E. pisi*. Molecular characterization of the ITS rDNA of *E. pisi* produced 650 bp nucleotides, PMITS (powdery mildew-internal transcribed region) primers produced 700 bp nucleotides, and an *Erysiphe* specific ITS primer pair amplified and synthesized 560 bp nucleotides. According to the findings, the collected *E. pisi* strains exhibited a low level of genetic diversity and only a slight differential in virulence on the host. In the study, *E. pisi* isolates from Anumapuram, Emerald Valley, Indira Nagar, and Thuneri showed a greater disease incidence in the natural field conditions and shared the same genetic lineage with other isolates in UPGMA hierarchical cluster analysis based on RAPD markers. There was no evidence of a link between the occurrence of the disease and these grouped populations.

## 1. Introduction

According to the Legume Taxonomy Working Group, the family Leguminosae (nom. alt. Fabaceae) is the third most diverse and socio-economically influential flowering family. It is a single monophyletic group that includes highly proteinaceous beans, legumes, and peas. There are over 800 genera and 23,000 species in this group, and they have been climatically resilient, cosmopolitan in distribution, and cultivated worldwide [1]. Along with cereals, legumes are important crops in providing dietary and livelihood needs for people. In addition to enhancing the physical, chemical, and biological characteristics of the land, legumes fix nitrogen in the soil [2,3].

Pea (field, garden, or green pea) is a widely domesticated winter crop of the Leguminosae family consumed as a pulse and a green vegetable. *Pisum fulvum* Sibeth. & Sm. and *Pisum sativum* L. are crucial cultivated species for fixing atmospheric nitrogen in the soil through a symbiotic relationship with *Rhizobium* bacteria. Due to its high protein content, the pea crop is an ideal animal feed and a suitable meat substitute for human nutrition. Mendel’s discovery of inheritance laws used the *P. sativum* (2n = 2x = 14) as a model species, providing the framework for modern plant breeding, cytology, and genetics [2]. However, progress in pea pathogenomics, or the gene-for-gene hypothesis with its parasites and genetic susceptibility to pathogens, has lagged considerably behind many other plant–pathogen interactomes. Peas are susceptible to various fungal pathogens and parasites, resulting in low production and productivity [4]. The fungal parasites move from one place to another by infectious conidia carried by the wind and sometimes with seeds and soil. This characteristic has resulted in significant contamination of pea crop breeding and domestication, and it is a highly effective technique for infecting crops shortly after seed germination. The most common disease of *P. sativum* is powdery mildew, which causes economic losses on a global scale [5]. The main breeding objective is to develop cultivars resistant to the predominant powdery mildew disease in specific geographic regions. *P. fulvum* is a new source of resistance in cultivated peas against powdery mildew and bruchid pest. Also, an intensive breeding program is needed for the wild species, *P. sativum* sub sp. *elatius* (Bieb.) Aschers. & Graebn., to develop resistant pea genotypes [2]. Disease control in peas by intrinsic plant resistance avoids the need for expensive fungicides, which may pose actual or perceived risks to the environment and humans. Employing resistant cultivars is the most effective technique for controlling phytopathogens. As for resistant varieties, there are limited choices, and their resistance depends on a very narrow genetic rationale. Only two recessive genes, *er1*, *er2*, and the single dominant gene, *Er3*, are being extensively exploited using linked markers in breeding programs worldwide [3,6]. As a result, host resistance to plant diseases such as powdery mildew is critical for long-term pea crop productivity.

Pea powdery mildew is an airborne fungal disease caused by *Erysiphe pisi* DC (Order: *Erysiphales*), a filamentous fungus responsible for 25–70% of yield losses in pea cultivation [7,8]. Recently, the authorized Global Biodiversity Information Facility (GBIF), Denmark, proposed the preferred name of the pathogen as *Erysiphe pisi* var. *pisi* DC. Furthermore, species such as *Erysiphe trifolii* Grev. or *Erysiphe baeumleri* U. Braun and S. Takam., in addition to *E. pisi*, may affect peas in specific environments [3,9]. However, the symptoms of powdery mildew caused by these pathogenic species are almost too similar to distinguish between them. There is considerable evidence that this disease impacts several important agronomical traits such as yield and quality [10]. It is also more damaging, especially to late-sowing or late-maturing cultivars [11]. Powdery mildew symptoms can emerge at the early stage of crop growth and are widespread in locations with warm, hot weather and cool nights. Infected plants may show as patches or be spread across a field. A white powdery mycelium and spores are visible in the early stages on leaves, pods, and stem surfaces. As the disease progresses, patches of grey-white mycelia cover the foliage completely [12]. The mycelia produce solitary, unbranched conidiophores and produce cylindrical wind-borne conidia. They form a single-celled, multilobed appressorium on the surface of plants. Haustoria are smooth, elliptic to round in shape, and formed within plant epidermal cells [13]; since *E. pisi* is naturally an obligate biotrophic fungus, only minimal information about its genotypic diversity, epidemiology, or the importance of population dynamics is available in the Nilgiris District of Tamil Nadu.

In pulse crops, rapid and accurate pathogen identification or disease diagnosis is essential for providing a suitable recommendation for sustainable disease management. Conventionally, powdery mildew species are identified based on the morphological characteristics of the anamorphic conidia and teleomorphic chasmothecia over the infected plant surface [14]. Due to its obligatory parasitic nature, the powdery mildew fungus cannot be grown on artificial media for identification [15]. Accurate detection of pathogenic fungal species is more complex due to sexual and asexual transitions and the diversified pathogenic strains or races spread worldwide. The diversity and variability within these species were determined in the previous studies using nrRNA internal transcribed spacer (ITS) sequences and teleomorphic characteristics, such as the chasmothecial appendage anatomy. However, it is difficult, time-consuming, and inappropriate when two species co-infect the same plant or when the teleomorphic stage is unusual in the area. Due to the extensive environmental impacts on growth and parasitism, it is challenging to determine the diversity of this fungus entirely based on morphological characteristics.

Molecular markers are becoming increasingly relevant for studying taxonomic and evolutionary relationships among various fungi [16]. These markers are ideal for screening and mapping the pathogen that causes powdery mildew in peas in a particular area. As a result, a few studies on the genetic diversity of *E. pisi* have been concerned and demonstrated the applicability of molecular markers for diagnosing and analysing *E. pisi* diversity. The internal transcribed spacer region (ITS) of fungi has been sequenced routinely. It has been confirmed to assist in developing molecular systematics at the species level and even within a species [17]. In addition, the constraint of identifying fungal strains based on a few morphological features can be overcome by using DNA-based techniques such as Amplified Fragment Length Polymorphism (AFLP), Internal Transcribed Spacer-Restricted Fragment Length Polymorphism of the rDNA (ITS-RFLP), Random Amplified Polymorphic DNA Polymorphism (RAPD), Universal Rice Primer (URP-PCR) and other relevant markers [16]. Among these is the RAPD technique, which has been demonstrated in various fungal species to investigate genetic variation [18,19]. In this context, single spore isolates of *E. pisi* from pea fields in 10 different places in the Nilgiris district of Tamil Nadu were molecularly characterized using the ITS region and the RAPD marker used to reveal its genetic diversity. Based on the aspects mentioned above, the purpose of this study is to determine the genetic diversity of pea powdery mildew fungus (*Erysiphe* sp.) in the Nilgiris as well as to correlate any differences between isolates in terms of physiological, virulence, and regional variability by employing both specific PCR amplification and RAPD marker analysis.

## 2. Materials and Methods

### 2.1. Collection of Isolates and DNA Extraction

We collected pea powdery mildew diseased leaves from ten growing villages in the Nilgiris ecosystem to study the phenotypic and genotypic characteristics of the pea powdery mildew pathogen. Then, the anamorphic propagules of isolates, including mycelium, conidiophores, and conidia, were separated from the infected leaves under aseptic conditions. These isolates were designated based on the regions, Anumapuram (P-ANU1), Emerald Valley (P-EMV1), Indira Nagar (P-IDN1), Thuneri (P-TNI1), Iduhatty (P-IHY1), Kenthorai (P-KTR1), Bigatti (P-BGI1), Palada (P-PLD1), Coonoor (P-CNR1), and Kotagiri (P-KGI1), for further studies (Figure S1). The collected isolates were observed under a microscopic image analyzer (LaboMet) at 400× magnification for further morphometric confirmation. Using the CTAB (cetyltrimethylammonium bromide) method, genomic DNA was isolated from anamorphic conidia of individual isolates [16] and further purified with Ribonuclease A (RNase A) catalyst (10 mg/mL) [20]. A nanodrop ND-1000 spectrophotometer was used to confirm the presence of genomic DNA (NanoDropTechnologies, Inc., Wilmington, DE, USA). Based on the spectrum interpretations, DNA was diluted in sterile milli-Q water to make an ultimate volume of 50 ng/μL and kept at −20 °C.

### 2.2. Molecular Characterization of E. pisi Using ITS Region-Specific Primers

*E. pisi* isolates were established at a genus level using ITS-1 and ITS-4, and the 5.8S ribosomal DNA (rDNA) region of the genus was amplified using ITS-1 and ITS-4 universal forward and reverse primers [17]. Then, the powdery mildew fungal internal transcribed spacer specific-primers viz., PMITS-1 (5′ TCGGACTGGCCYAGGGAGA 3′) & PMITS-4 (5′ TCACTCGCCGTTACTGAGGT 3′) [13] and EryF (5′-TACAGAGTGCGAGGCTCAGTCG-3′) & EryR (5′-GGTCAACCTGTGATCCATGTGACTGG-3′) [18] were used for further confirmation of *E. pisi* isolates. The PCR amplification was performed as per the protocols cited [18] using an Agilent PCR master cycler and then documented the banding profiles of PMITS and *Erysiphe*-specific PCR products with the comparison of 1 kb marker already loaded on the gel along with the samples.

### 2.3. Genetic Discrimination by RAPD Marker

Twenty random amplified polymorphic DNA (RAPD) oligo-primers (Chromous Biotech, Bangaluru, India) were used to determine the genetic variability among the ten isolates of *E. pisi* (Table 1). The 20 μL PCR reaction mixture prepared for each isolate contains a 25 ng DNA template, 10× Taq buffer, 2.5 mM of dNTP mixture, 2.5 mM of MgCl_2_, 30 pmol of random primer, and three units of Taq DNA polymerase (Genei, Bangaluru, India) separately. Using a thermocycler (Eppendorf Mastercycler gradient, Westbury, NY, USA) amplification was performed under the following cycles: initial denaturation at 94 °C for 5 min, followed by 30 cycles of denaturation at 94 °C for 1 min, annealing at 36 °C for 1 min, extension at 72 °C for 2 min, and final elongation at 72 °C for 5 min. The amplified PCR products were subjected to a 1.5% agarose to separate the amplified products by primer sequence. Subsequently, gels were then individually illuminated with a UV transilluminator and photographed using the BioRad gel documentation system

### 2.4. Band Scoring and Data Analysis

After repeating the RAPD-PCR experiment three times, only the consistently randomly amplified bands that emerged were evaluated. We started with the most considerable fragment and counted the banding patterns of each RAPD primer, moving down to the smallest fragment. Each band present or absent in each isolate was assigned a 1 or 0. A similarity matrix analogue was created to disclose genetic relationships using a statistical tool called Plymouth Routines in Multivariate Ecological Research (PRIMER v7) by Quest Research Limited, New Zealand. This multivariate analysis was produced by the Unweighted Pair Group Method with Arithmetic Mean (UPGMA). It evaluated the similarities, dissimilarities, and distances using hierarchical cluster analysis (HCA) with the Bray–Curtis coefficient. Non-metric multidimensional scaling (NMDS) was used to scale unorganized *E. pisi* populations.

### 2.5. The Efficacy of RAPD Primers for Evaluating the Diversity of E. pisi

The efficacy of primers in determining the genetic variability of *E. pisi* isolates was studied based on its polymorphism. We quantified the total number of bands, the number of monomorphic and polymorphic bands, the number and frequency of bands in each primer, and the ratio of polymorphic to monomorphic bands. In addition, we applied the developed formula to calculate Polymorphic Information Content (PIC).

PICj = 1 − ∑l = 1 to L P2lj

where P2lj denotes the relative frequency of the locus j’s first allele and is constant across all alleles (L) at all loci. The MI (Marker Index) was calculated as the measure of PIC and the frequency of polymorphic bands per test unit; in comparison, the EMR (E) is calculated as the average of polymorphic loci segments and the total number of polymorphic loci for an individual test.

EMR (E) = np (np/n)

where ‘np’ denotes the number of distinct polymorphic loci and ‘n’ represents the total number of loci.

## 3. Results and Discussion

### 3.1. Disease Symptoms

**Figure 1:**
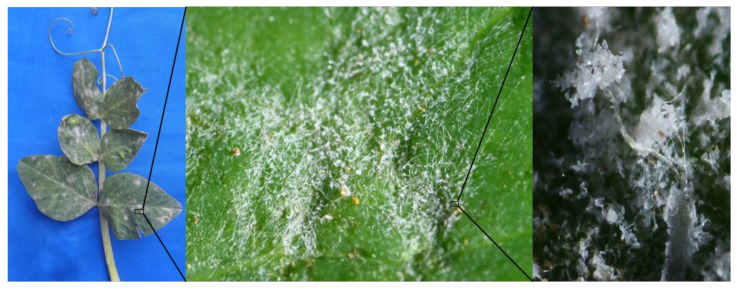
Powdery mildew symptoms showing aerial mycelium and conidia on pea leaves.

*Erysiphales* is a taxonomic order of ascomycetous fungi that cause powdery mildew disease with a nearly global distribution. This order consists of 18 genera and about 900 species [21]. *Erysiphales* is the most common parasitic fungus and affects over 10,000 plant species, including many vital to the economy [22]. *Erysiphe* is the largest, compared to additional teleomorphic genera, and accounts for about 50% of powdery mildew genera. Powdery mildew species develop within plant tissue and are generally epiphytic, forming symptomatic white mycelial mats on almost every plant organ [4]. Numerous studies on *Erysiphe* spp. have already been conducted in various geographical areas. Most often, abundantly produced asexual spores are spread by the wind and cause the infection. Occasionally, a sexual recombination process develops more virulent strains for widespread adaptability to varied habitats [23]. Due to the obligatory parasitic and nonculturable nature, most studies on powdery mildew biodiversity in a region rely on local surveys and samplings. In this study, powdery mildew samples were collected in the Nilgiris district, and we observed heavy infection pressure in all the sampled sites. Visual symptoms include minute, diffused, irregular, and abundant patches of white-to-greyish talcum powder-like fungal growth on the upper epidermal leaf surface of the lower leaves and stipules (Figure 1). The lesions quickly spread throughout the plant, covering the stem, tendrils, and florets with a filmy white growth. Powdery mildew signs were found mainly on stems in later infections rather than on leaves in these regions. The fungus infects the pods at the severe stage and forms minute black fruiting structures within the spores. In the Nilgiris district of Tamil Nadu, where the pea is widely grown throughout the year, pea powdery mildew, caused by *E. pisi*, is a severe and recurring disease. According to Bahadur et al., 2008 [24], pea powdery mildew is one of the most virulent and widespread diseases affecting both primary and off-season peas in this region. The disease spreads to epidemic levels almost every year in diverse hilly places with warm, dry seasons and cool nights, where the crop is cultivated all year. This *E. pisi* fungal parasite penetrates chloroplast-deficient epidermal cells through haustoria. Furthermore, initial mycelial colonization starts on basal mesophyll cells and then produces superficial mycelia over the plant surface, which interrupts the photosynthetic activity, and a severe outbreak can alter total biomass, plant height, pods per plant, seed yield, and node formation, resulting in crop death and production losses of up to 50% [25]. In India, the disease incidence was recorded at 40% in the Chandel and Imphal districts of Manipur state, and the extreme incidence in Pant vegetable pea (94% PDI) and Azad-P-3 (90% PDI) at the 85th day of the old crop [26].

### 3.2. Morphological Characterization of E. pisi

**Figure 2:**
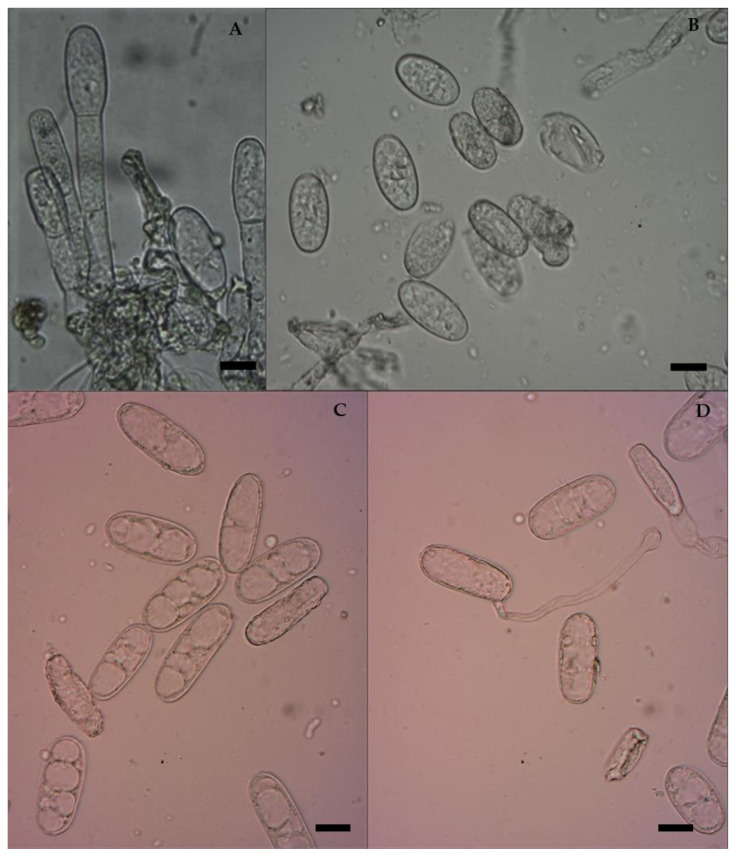
Micromorphological characters of *E. pisi*. Scale bars = 10 µm. (**A**) Germinating conidiophore of *E. pisi*; (**B**) Young conidia; (**C**) Matured conidia; (**D**) Germination of conidia producing appressorium.

The traditional taxonomic system of powdery mildew fungi relies on morphological characteristics to classify specific forms and races. A microscopic image analyzer was used to perform morphological characterization and particular measurements of the conidia. The conidia of *E. pisi* were studied for characteristics such as colour, shape, and size. The observations showed that the fungal hyphae were superficial, ectophytic, thin-walled, septate, and branched over the leaf surface, anchored with the support of absorptive haustoria. Conidiophores are cylindrical, with three to four cells, and produce chains of hyaline, single, thin-walled, immature ellipsoid-ovoid to mature dolliform (cylindrical) conidia (Figure 2), whereas more typically ellipsoid–dolliform conidia are distinctive for *E. pisi* [27]. The mycelium, conidiophores, and asexual conidia of the collected powdery mildew samples were reliable with that of *E. pisi*. These characters matched those in early descriptions of *E. pisi* on peas [10,11]. All isolates were maintained in a glasshouse under natural host circumstances, and their morphological traits were monitored regularly. Naturally, conidia of *E. pisi* which land on the leaves develop solitary, dome-shaped, multilobed, and melonized appressoria that extend as infection pegs and penetrate the epidermal host tissues through the cuticle and cell wall. Then, the parasitizing haustoria which develop beneath the crop epidermis are smooth and ovoid to spherical [4,11,28]. The production of haustoria is abundant in susceptible cultivars and relatively ineffective or produces hypersensitization in resistant genotypes, which could quickly halt haustoria proliferation. These entities are not visible beneath the appressoria in the host tissues [19]. To date, the first and only *Erysiphe* species believed to be responsible for the powdery mildew in most legume crops, including alfalfa and peas, was *E. pisi*, which is morphologically identical, but taxonomically divided into three *formae speciales* based on the host, namely, *E. pisi* f. sp. *medicaginis* Boerema & Verhoeven, *E. pisi* f. sp. *pisi* DC., and *E. pisi* f. sp. *vicia-sativa* Boerema & Verhoeven [4]. While various *Erysiphe* species have been used to study essential components of host–parasite relationships, morphology, histopathology, and molecular biology, there are still numerous uncertainties regarding species validation. New reports and discussions have revealed that *Erysiphe* species in legumes are more complex than previously recognized. Their systematics are still undergoing significant revisions [4,22].

### 3.3. Molecular Characterization of Isolates of E. pisi

**Figure 3:**
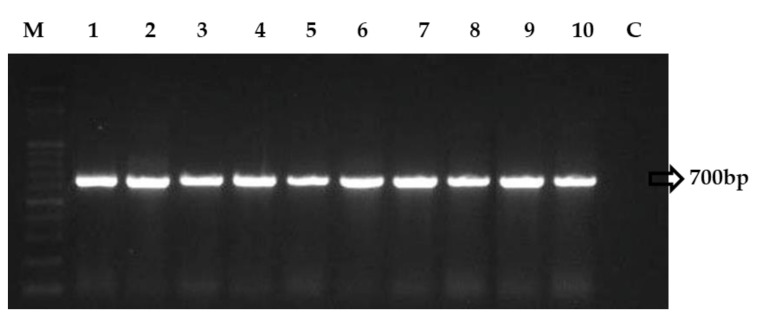
PCR amplification of PMITS region of the isolates of *E. pisi.* (M—Marker, 1—Anumapuram (P-ANU1), 2—Emerald Valley (P-EMV1), 3—Indira Nagar (P-IDN1), 4—Thuneri (P-TNI1), 5—Iduhatty (P-IHY1), 6—Kenthorai (P-KTR1), 7—Bigatti (P-BGI1), 8—Palada (P-PLD1), 9—Coonoor (P-CNR1), 10—Kotagiri (P-KGI1) and C—Control).

**Figure 4:**
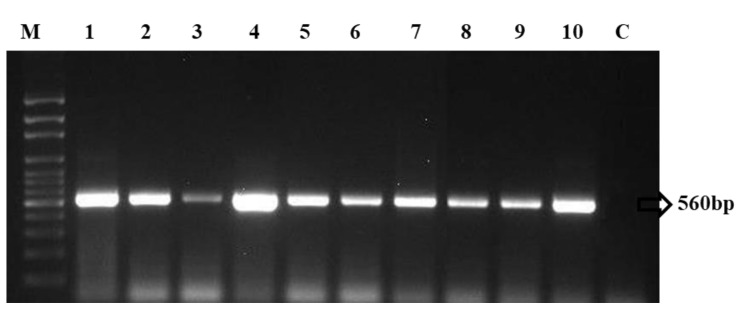
PCR amplification of *Erysiphe* specific ITS primer for the isolates of *E. pisi.* (M—Marker, 1—Anumapuram (P-ANU1), 2—Emerald Valley (P-EMV1), 3—Indira Nagar (P-IDN1), 4—Thuneri (P-TNI1), 5—Iduhatty (P-IHY1), 6—Kenthorai (P-KTR1), 7—Bigatti (P-BGI1), 8—Palada (P-PLD1), 9—Coonoor (P-CNR1), 10—Kotagiri (P-KGI1) and C—Control).

Precise identification is necessary for developing effective long-term management strategies in field conditions. Nevertheless, these obligate biotrophs create challenges for pathologists since many *Erysiphe* species lack distinct morphological features and are impossible to culture artificially, necessitating molecular approaches for differentiation. Traditional diagnostic methods rely on a correlation between host identification and associated pathogen morphology. However, genomic rDNA regions have been analysed to overcome unpretending identification obstacles in fungal pathogens [29]. The genetic studies, which included ITS and 28S rDNA regions and examination of morphological features, provided the resolution of species distinction within a polymorphic complexity. Ten isolates were amplified in this study using the ITS universal primers ITS-1 and ITS-4, and the results indicated that all isolates appeared to contain the predicted amplicon of 650 bp. Following that, PCR amplification using the PMITS primer pair (PMITS1 and PMITS2) and the *Erysiphe*-specific ITS primer pair (EryF and EryR) revealed an amplicon of 700 and 560 bp, respectively (Figure 3 and Figure 4). The ITS gene region of fungal DNA is critical for both species and intra-species molecular systematics. The variation between individual rDNA repeats can occasionally be observed amongst the ITS and IGS regions. The amplicon size of the 18s rRNA region-based primers, *viz*., ITS 1 and 4, PMITS 1 and 2, and EryF and EryR on *E. pisi* consistent with the reported nucleotide size of *E. pisi*, from central and northeast India [8]. These findings confirmed the identity of all of the isolates investigated. As a result of this finding, the molecular ITS region could not detect any significant variation within the *E. pisi* isolates. Instead, it grouped them all into a single line. Another study showed that such molecular techniques are appropriate and feasible in phylogenetically closely related powdery mildew species assemblages for which ITS investigations do not have widespread value [30]. It has been found that when populations get more geographically distant, the discrimination capacity of this technique declines. Analysis of the internal transcribed spacer (ITS) region of ribosomal RNA genes is a widespread technique for describing the diversification among fungal communities due to its versatility for describing this fungus at the genus or species level and its many essential characteristics, such as its predominance in available genomic sequences. In addition, this technique offers several essential characteristics for analyzing the genetic diversity of both prominent and subpopulations [14].

### 3.4. The Genetic Diversity of E. pisi in the Nilgiris

**Figure 5:**
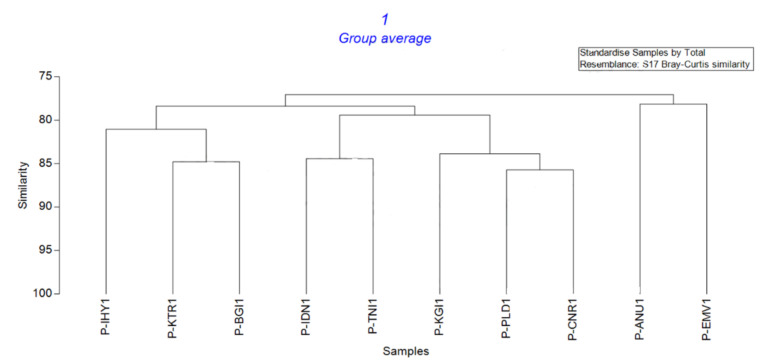
UPGMA cluster analysis of *E. pisi* based on RAPD primers. (P-IHY1—Iduhatty, P-KTR1—Kenthorai, P-BGI1—Bigatti, P-IDN1—Indira Nagar, P-TNI1—Thuneri, P-KGI1—Kotagiri, P-PLD1—Palada, P-CNR1—Coonoor, P-ANU1—Anumapuram, P-EMV1—Emerald Valley).

Molecular markers are typically utilized to differentiate the evolution of parasitic fungus, particularly about the potential to assimilate resistant genotypes, diverse habitats, and fungicides. Most research on the diversity and variability of *E. pisi* infecting peas has relied on RFLP and RAPD markers [31]. RAPD primers produced 162 banding patterns from the ten isolates investigated in this study. The twenty primers used in the study showed significant polymorphism within the fungus. Compared to the other primers studied, OPF10 produced more banding patterns of 14 and has a PIC value of 0.931 and an EMR of 13.22 (Table 2). Regarding the RAPD fingerprinting, the HCA-based UPGMA cluster revealed a 78–88% similarity matrix index across *E. pisi* isolates based on the Bray–Curtis similarity index. P-ANU1 Anumapuram and P-EMV1 Emerald Valley isolates were found to be closer, with 78.15% similarity. A dendrogram generated by cluster analyses indicated two distinct major groupings, demonstrating the genetic linkages of isolates. P-PLD1-Palada and P-CNR1-Coonoor isolates were 85.71% genetically identical when grouped with the other isolates. The cluster analysis based on genetic similarity coefficients is depicted in Figure 5.

**Figure 6:**
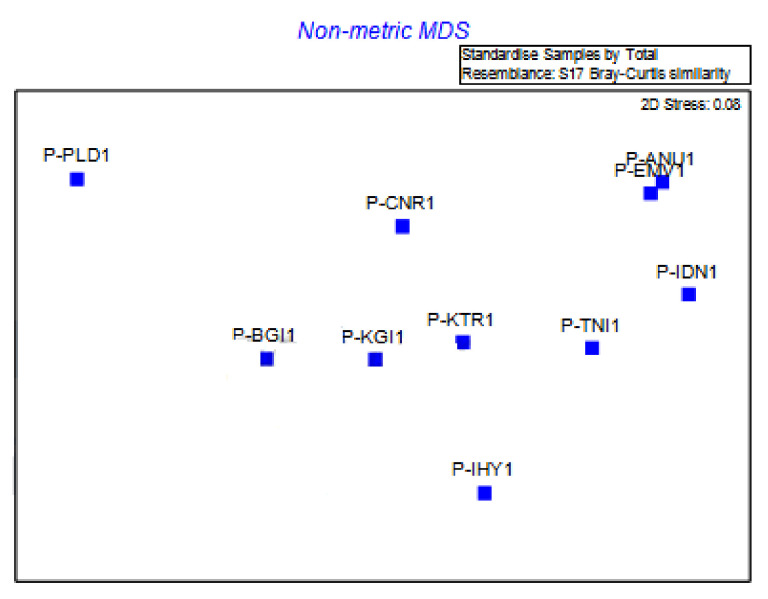
Non-metric MDS analysis of *E. pisi* isolates based on RAPD primers. (P-IHY1—Iduhatty, P-KTR1—Kenthorai, P-BGI1—Bigatti, P-IDN1—Indira Nagar, P-TNI1—Thuneri, P-KGI1—Kotagiri, P-PLD1—Palada, P-CNR1—Coonoor, P-ANU1—Anumapuram, P-EMV1—Emerald Valley).

Using RAPD marker fingerprints, the indirect gradient analysis was done by 2D non-metric multidimensional scaling (NMDS) based on the Bray–Curtis similarity index, which accurately distinguished individuals of *E. pisi* (Table 3). This scaling sorts out the relational patterns among genetic factors (gene regions) from time-series data. The ten isolates analysed produced two massive geographic clusters and one single geographic cluster (Figure 6). As per the study, the collected *E. pisi* strains have been found to have a small amount of genetic diversity and slight variation in virulence on the host.

In most genetic and taxonomic studies of different fungi, nucleic acid-based molecular markers, such as Random Amplified Polymorphic DNA (RAPD), have been utilized to characterize the pathogen’s diversity in the study area. The ability of a pathogen to adapt to its environment is reflected in its genetic diversity. As a result, the degree of pathogenic variation would contribute to determining the degree of adaptability. Our research discovered a substantial genetic variability in *E. pisi*, leading us to conclude that the pathogen has evolved the ability to adapt to both the macro- and micro-environments to which it is exposed during pea cultivation. The RAPD primers revealed the genetic variation among *E. pisi* isolates through the amount of polymorphism. In addition, dealing with an obligate parasite such as *E. pisi* challenges the selection procedure for developing powdery mildew resistance. To overcome these challenges, most RAPD markers studied were linked to finding powdery mildew resistance genes, which may play a prominent role in recognizing resistance loci and pyramiding resistance genes in various pea cultivars [23].

In the present research, RAPD primers produced many polymorphic bands, indicating pairwise genetic distances in the genomic region targeted by the primers possessed a high level of variation. P-ANU1, P-EMV1, P-IDN1, and P-TNI1 isolates were collected from geographically nearby locations with a greater incidence of disease in the field and belonged to the same genetic lineage as other isolates. There was no indication of a link between the disease occurrence and these populations in the region. According to the study’s findings, the RAPD marker is more useful for defining small pathogenic clade or closely related clade with sequence similarities. As a result, the RAPD marker can be used to determine how much *E. pisi* genetic variation occurs within a specific region in a particular period, further validating its use in marker-assisted selection [19].

Over many generations, powdery mildew isolates subjected to ongoing evolutionary pressures, either with efficient vertical resistance genes or fungicides, might demonstrate considerable adaptability. In this study, it was evident that low-level genetic variability among the pathogens, favourable climatic conditions for pathogen infection, and selective host parasitism on peas in the region contributed to a wide range of potential influences for the rapid spread of pathogenic species. This work explored some powdery mildew-infested biogeographical regions that could provide valuable scientific insight to promote the resistant cultivars of those remaining locations. Additionally, this is the first investigation into the fungal diversity in the Nilgiris biosphere region. Furthermore, collecting additional powdery mildew samplings of peas from wider regions is desirable for better marker-assisted selection in future work.

## 4. Conclusions

Pea is the most widely cultivated pulse crop in the Nilgiris region of India. Diseases such as powdery mildew, however, have dampened its productivity. Powdery mildew, the most frequent field pea disease in the Nilgiris district, can be identified and characterized as a result of this research, which will aid in the detection and characterization of the disease. The use of polymerase chain reaction (PCR) for molecular investigations helps clarify the identification at the species level as well as the genetic diversity between isolates. Identifying appropriate and long-lasting control strategies is a massive obstacle to sustainable agricultural research in pea. Also, to maintain the lifespan of recently developed resistant cultivars in the region, it is crucial to understand the genetic variation of local fungal populations and the mechanisms that contribute to distinct *formae specialis*/strains. Understanding the degree and spread of genetic variation among pathogen populations assists in defining its ecology and designing efficient standard precautions against infection. Understanding evolutionary dynamics brings us closer to predicting disease population increases in agricultural habitats. This knowledge is beneficial for plant pathologists and breeders seeking to develop appropriate management tactics.

## Figures and Tables

**Table 1:** Primers used for RAPD analysis.

S. No.	Primers	Sequence 5′-3′
1.	OPA-01	CAGGCCCTTC
2.	OPA-03	AGTCAGCCAC
3.	OPA-05	AGGGGTCTTG
4.	OPA-07	GAAACGGGTG
5.	OPA-09	GGGTAACGCC
6.	OPA-18	AGGTGACCGT
7.	OPB-02	TGATCCCTGG
8.	OPC-08	TGGACCGGTG
9.	OPC-12	TGTCATCCCC
10.	OPE-01	CCCAAGGTCC
11.	OPF-01	ACGGATCCTG
12.	OPF-06	GGGAATTCGG
13.	OPF-10	GGAAGCTTGG
14.	OPF-12	GGCTGCAGAA
15.	OPF-14	TGCTGCAGGT
16.	OPG-05	CTGAGACGGA
17.	OPG-08	TCACGTCCAC
18.	OPG-11	TGCCCGTCGT
19.	OPG-16	AGCGTCCTCC
20.	OPL-05	ACGCAGGCAC

**Table 2:** Genetic diversity of the isolates of *E. pisi* revealed by RAPD analysis.

S. No.	Primers	Sum of Banding Pattern	Polymorphic Banding Pattern	Monomorphic Banding Pattern	PIC Value	EMR
1.	OPA-01	10	9	1	0.903	9.05
2.	OPA-03	6	5	1	0.822	5.38
3.	OPA-05	8	7	1	0.849	0.015
4.	OPA-07	7	6	1	0.820	7.80
5.	OPA-09	10	9	1	0.829	9.05
6.	OPA-18	4	3	1	0.749	3.56
7.	OPB-02	4	3	1	0.749	3.12
8.	OPC-08	10	9	1	0.944	13.94
9.	OPC-12	10	9	1	0.884	9.05
10.	OPE-01	10	9	1	0.800	8.10
11.	OPF-01	10	9	1	0.898	9.05
12.	OPF-06	10	9	1	0.899	9.05
13.	OPF-10	14	13	1	0.931	13.22
14.	OPF-12	6	5	1	0.830	9.20
15.	OPF-14	7	6	1	0.848	5.40
16.	OPG-05	7	6	1	0.855	5.93
17.	OPG-08	8	7	1	0.869	7.27
18.	OPG-11	7	6	1	0.821	5.14
19.	OPG-16	7	6	1	0.791	5.40
20.	OPL-05	7	6	1	0.786	5.14
	Total	162	142	20		

**Table 3:** Similarity matrix of the isolates of *E. pisi* generated by RAPD analysis.

Isolate	P-ANU1	P-EMV1	P-IDN1	P-TNI1	P-IHY1	P-KTR1	P-BGI1	P-PLD1	P-CNR1	P-KGI1
P-ANU1	100									
P-EMV1	78.15	100								
P-IDN1	79.83	80.36	100							
P-TNI1	77.87	73.77	84.43	100						
P-IHY1	78.15	74.58	76.27	78.69	100					
P-KTR1	76.80	76.80	76.00	79.20	81.60	100				
P-BGI1	78.99	79.13	80.00	79.51	80.51	84.80	100			
P-PLD1	77.18	76.37	76.37	81.48	77.19	79.52	79.69	100		
P-CNR1	74.62	73.08	76.15	83.85	76.92	83.08	76.92	85.71	100	
P-KGI1	78.13	77.34	75.78	82.81	75.78	81.25	75.78	82.35	85.39	100

## Data Availability

Not applicable.

## Supplementary Materials

The following supporting information can be downloaded at: https://www.mdpi.com/article/10.3390/jof8080798/s1, Figure S1: Collection sites.
