# Supplementary figures and images for: Genetic Diversity Analysis based on the Virulence, Physiology and Regional Variability in Different Isolates of Powdery Mildew in Pea
Source: J Fungi (Basel). 2022 Jul 29;8(8):798. doi: 10.3390/jof8080798 (PMC9409743; doi:10.3390/jof8080798)

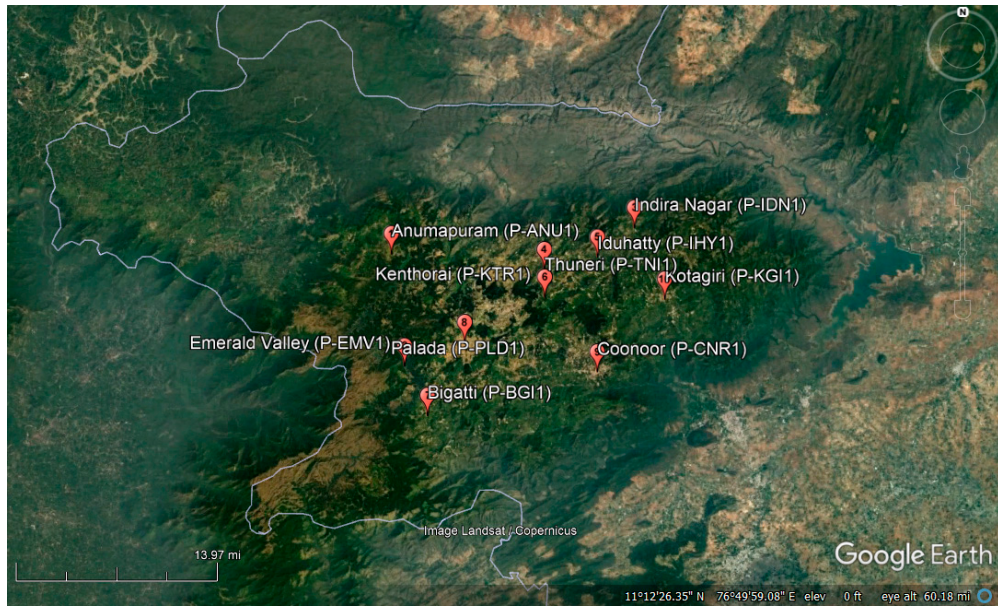

**Figure S1.** Collection sites (pinned in Google Earth Pro for non-commercial purpose).

Supplement: Supplementary file 1 [file jof-08-00798-s001.zip › jof-1728093-supplementary.pdf]
